# Reasons for SARS-CoV-2 infection in children and their role in the transmission of infection according to age: a case-control study

**DOI:** 10.1186/s13052-021-01141-1

**Published:** 2021-09-27

**Authors:** Mauro Calvani, Giulia Cantiello, Maria Cavani, Eleonora Lacorte, Bruno Mariani, Valentina Panetta, Pasquale Parisi, Gabriella Parisi, Federica Roccabella, Paola Silvestri, Nicola Vanacore

**Affiliations:** 1 Operative Unit of Pediatrics, San Camillo-Forlanini Hospital, 00151 Rome, Italy; 2 Rome, Italy; 3 Department of Maternal, Infantile and Urological Sciences, Sapienza University of Rome, 00161 Rome, Italy; 4 National Centre for Disease Prevention and Health Promotion, National Institute of Health, 00161 Rome, Italy; 5 Laboratory of Microbiology and Virology, San Camillo-Forlanini Hospital, 00151 Rome, Italy; 6 L’altrastatistica srl, Consultancy & Training, Biostatistics office, Rome, Italy; 7 NESMOS Department, Faculty of Medicine & Psychology, “Sapienza” University, c/o Sant’Andrea Hospital, Rome, Italy; 8 Child Neurology, NESMOS Department, Faculty of Medicine & Psychology, “Sapienza” University, c/o Sant’Andrea Hospital, Rome, Italy

**Keywords:** SARS-CoV-2 infection, Covid-19, Children, School contact, Household contact, Secondary attack rate

## Abstract

**Background:**

The locations where children get exposed to SARS-CoV-2 infection and their contribution in spreading the infection are still not fully understood. Aim of the article is to verify the most frequent reasons for SARS-CoV-2 infection in children and their role in the secondary transmission of the infection.

**Methods:**

A case-control study was performed in all SARS-CoV-2 positive children (*n* = 81) and an equal number of age- and sex- matched controls who were referred to the S. Camillo-Forlanini Pediatric Walk-in Center of Rome. The results of all SARS-CoV-2 nasopharyngeal swabs performed in children aged < 18 years from October 16 to December 19, 2020 were analyzed.

**Results:**

School contacts were more frequent in controls than in cases (OR 0.49; 95% CI: 0.3–0.9), while household contacts were higher in cases (OR 5.09; 95% CI: 2.2–12.0). In both cases and controls, school contacts were significantly less frequent, while on the contrary household contacts seemed to be more frequent in nursery school children compared to primary school or middle/high school children. A multivariate logistic regression showed that the probability of being positive to SARS-CoV-2 was significantly lower in children who had school contacts or who had flu symptoms compared to children who had household contacts. Results showed a 30.6% secondary attack rate for household contacts.

**Conclusion:**

In our study population, the two most frequent reasons for SARS-CoV-2 infection were school and home contacts. The risk of being positive was 5 times lower in children who had school contacts than in children who had household contacts.

**Supplementary Information:**

The online version contains supplementary material available at 10.1186/s13052-021-01141-1.

## Background

It is still unclear where children are exposed to SARS-CoV-2 infection and their contribution in spreading the infection. In most cases of COVID-19, the infection is acquired at home [1, 2] . However, the way SARS-CoV-2 infection is transmitted in other locations, such as schools, is still indefinite and mainly reported in studies referring to the first phase of the pandemic, when schools were mostly closed [3]. Children of all ages can be infected and can therefore spread the virus. Young children seem to have a lower susceptibility to infection compared to adults, with individual susceptibility increasing with age [4, 5]. Some studies suggest that children < 10 years may have a relatively small role in the transmission of the infection [6, 7]. However, it is still unclear whether children are able to transmit SARS-CoV-2 as much as adults, [8] and most of the available information comes from studies carried out on symptomatic children: generally the literature suggests that children do not seem to be particularly contagious [ 9].

The first SARS-CoV-2 outbreak was described in Wuhan, China, in December 2019 [ 10]. On February 20, 2020, patient 1 was diagnosed in Italy. Schools of all levels were closed on March 4, and lockdown measures were implemented on March 11, in an attempt to contain the spread of the COVID-19 pandemic [11]. Thanks to preventive measures during the summer season COVID-19 infection decreased in Italy. Schools reopened, between the 14th and the 24th September 2020 [12]. At the end of September, the number of cases has increased again (the second wave) peaking in December 2020. Since then there has been a slow reduction in cases until March 2020 when it had a further slight increase peaking in May 2020 followed by dramatic fall and finally reducing a lot, thanks to the vaccines implementation and the arrival of the new summer season [13]. Finally, during these last days, the appearance of more widespread variants is again increasing the number of infections. A similar path has been observed in other countries [14].

Although the use of Pfizer-BioNTech COVID-19 vaccines has been authorized and recommended in adolescents aged 12–15 years [15], only a few children in Italy have been vaccinated and there is still discussion in the scientific literature and on social media about the advisability of performing it and the uncertainty in the population [16–18].

All these reasons might lead to a spreading situation of the virus within the next months, especially during the fall when kids will be back in schools and people will be indoors, so it can be useful to acknowledge the reasons for SARS Cov-2 infection in children and their role in spreading the infection.

## Methods

The main aims of the present study were to identify the most frequent reasons for SARS-CoV-2 infection during the second wave of the pandemic, and the role of children in the secondary transmission of the infection. The secondary aims included verifying the type and frequency of symptoms in symptomatic children, and the adherence, at home, to preventive measures to limit the spreading of SARS-CoV-2 in children from 3 different age groups, nursery school, primary school, and middle/high school.

The results from all SARS-CoV-2 nasopharyngeal swabs (NS) performed in children and adolescents aged < 18 years at the S. Camillo-Forlanini Pediatric Walk-in Center (SCPWC) from October 16 to December 19, 2020 were analyzed. According to the Italian Health Service of Lazio Region, Central Italy [19], all children with a suspected SARS-CoV-2 infection were administered an antigen rapid detection test (Ag RDT) and, if appropriate (positive Ag RDT with cut off index (COI) < 10 UI), a SARS-CoV-2 nucleic acid amplification test (NAAT). Children were diagnosed as positive for SARS-CoV-2 in case of positive Ag RDT (COI > 10) or when an Ag RDT < 10 UI was confirmed by a positive SARS-CoV-2 NAAT result [20]. Children with a negative SARS-CoV-2 antigenic test or with an antigenic test < 10 UI and a negative NAAT test result were classified as negative. All children who were diagnosed as positive for SARS-CoV-2 infection and an equal number of age- and sex-matched controls were enrolled in this case-control study. The first age- and sex- matched consecutive child who was classified as negative was selected as control.

Parents were contacted and interviewed by telephone using a standardized questionnaire (Fig. 1 in Supplemental content). The Italian school system is structured in 4 levels: nursery school (0–5 years), primary school (6–10 years old), middle/junior high school (11–13 years), and high school (14–18 years). Nursery, primary, and middle/junior high schools were opened during our study period, while high schools were opened only until October 26th, when remote teaching was activated [3]. We classified children into 3 age-groups: nursery school (0–5 years), primary school (6–10 years), middle/high school (11–18 years). Four trained interviewers administered structured questionnaires by telephone.

**Fig. 1:**
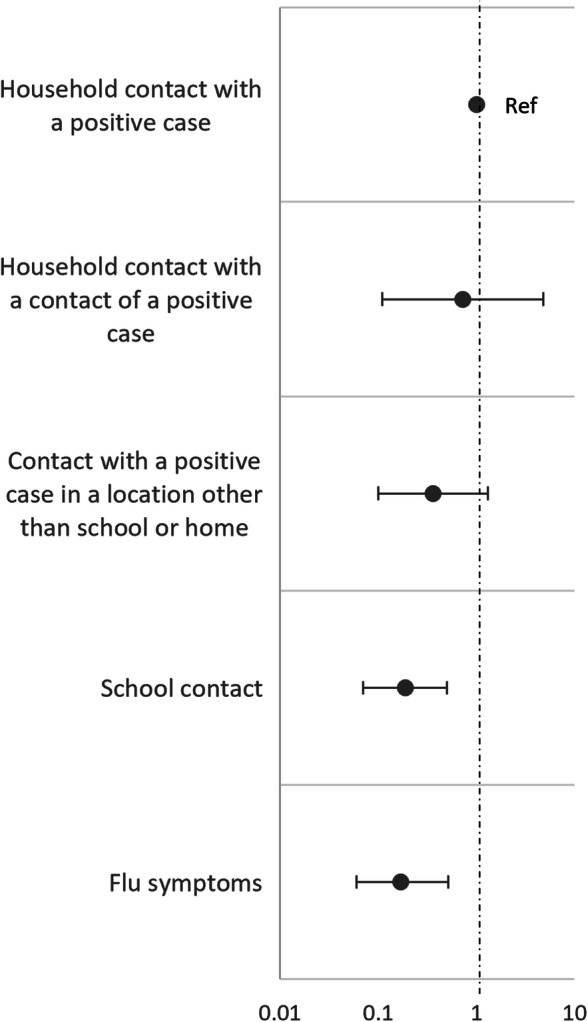
Unconditional multivariable logistic regression with robust errors in children that reported only 1 reason for undergoing a NS (*n* = 147)

T test was used to compare age between positive and negative children in the tested population. Chi square or Fisher exact tests were used to compare data between groups. In case of comparisons between the three age-classes, the chi square for trend was also reported. Univariable logistic regressions were used to calculate the Odd Ratio (OR) and 95% confidence interval (95% CI) of a positive result for each reason of NS in cases compared to controls.

A multivariable logistic regression with robust errors was used to assess the influence of the reason for undergoing a NS on the probability of being positive for SARS-CoV-2. Considering that multiple reasons were reported only in positive children, and thus some events are completely determinate (quasi complete separation), the multivariable logistic regression was applied only to cases and controls who reported one single reason. The reason was treated as dummy variable, and the positivity of a family member was considered as a reference value. Adjusted odd ratio (aOR) and their 95% CI were calculated. A sensitivity analysis was performed with: 1) multivariable logistic regression with paired data (age and sex) as adjusting variables, 2) multivariable logistic regression with clusters (pairs), 3) conditional logistic regressions (*n* = 132) (Table 1 Supplemental content). The secondary attack rate and its exact Clopper Pearson 95% CI was also calculated. The software Stata 16.1 was used for all analysis, and a *p* value < 0.05 was considered as statistically significant.

## Results

During the study period, 2837 children were referred to the SCPWC, 52% of whom were males, with a mean age of 8.7 (SD 4.3) years. Of these, 96 (3.4%) children were diagnosed as positive for SARS-CoV-2. No significant differences were observed in sex and age between positive and negative children (Table 1). All telephone questionnaires were administered within 2 months from the NS test. Some parents could not be contacted or refused to be interviewed, thus reducing the population to 81/96 (84.3%) positive cases. Therefore, we considered as our study population 162 children (81 SARS-CoV-2 positive and 81 Controls), of whom 54.3% were males, 46 went to nursery school, 62 to primary school, and 54 to middle/high school. (Table 2 in Supplemental content).

**Table 1:** Demographic data of all children that underwent a SARS-CoV-2 NS in the SCPWC

	All		SARS-CoV-2 positive		Controls		*P*
N	2837		96		2741		
**Sex (n,%)**							
Male	1468	51.7	50	52.1	1418	51.7	0.946
Female	1369	48.3	46	47.9	1323	48.3	
**Age**							
Mean (sd)	8.7	4.3	8.52	4.52	8.6	4.5	0.798
Nursery school (n,%)	800	23.2	28	29.2	772	28.2	0.800
Primary school (n,%)	1061	35.1	38	39.6	1023	37.3	
Middle/high school (n,%)	976	41.7	30	31.3	946	34.5	

Nursery school: 0–5 years; Primary school: 6–10 years; Middle/high school: 11–19 years

Regarding the first aim of the study, the main reasons for being referred to the SCPWC for a NS in the whole study population were school contacts in 70 (43%) children, the presence of a SARS-CoV-2 positive family member in 37 (23%) children, the presence of flu symptoms in 36 (22%) children, having been in contact with a SARS-CoV-2 positive subject in a different location than school or at home in 21 (13%) children, and the presence of a family member who had been in contact with a SARS-CoV-2 positive subject in 14 (9%) children. Continuing the investigation on the first objective within the two study groups, in cases who reported school contacts, the reported contact was more frequently a SARS-CoV-2 positive child (*n* = 33) rather than a teacher (*n* = 23). School contacts were more frequent in controls (51.9%) than in cases (23.6%)(*P* = 0.02), while a household contact with a positive subject or with a contact of a positive subject was more frequent in cases than in controls (35.8% vs 9.9%) (*P* = 0.001) (Table 2).

**Table 2:** Reasons for performing a SARS-CoV-2 NS

Reasons	SARS-CoV-2 positive		Controls		OR	95% CI	*P*
	N	%	n	%			
N	81		81				
School contact (all)	28	34.6	42	51.9	0.49	0.3–0.9	0.027
Contact with a positive teacher	4	4.9	19	23.5	0.17	0.1–0.5	0.002
Contact with a positive child	10	12.3	23	28.4	0.36	0.2–0.8	0.013
Flu symptoms	15	18.5	21	25.9	0.65	0.3–1.4	0.259
Household contact with a positive case	29	35.8	8	9.9	5.09	2.2–12.0	< 0.001
Household contact with a contact of a positive case	12	14.8	2	2.5	6.87	1.5–31.8	0.014
Contact with a positive case in a location other than school or home	13	16.0	8	9.9	1.74	0.7–4.5	0.246

Significant differences were observed in the reasons for being referred to the SCPWC for a NS across the three age groups, with school contact being less frequent in nursery school children (23.9%) compared to primary school (51.6%) and middle/high school children (50%) (*p* = 0.008). The probability of having had a contact with a positive child at school increased significantly with age, with a 6.5% probability in nursery school, a 22.6% probability in primary school, and a 29.6% probability in high/middle school (*p* = 0.005). Family contact, resulted more frequent in nursery school children (37.0%) compared to primary school (19.4%) and middle/high school children (14.8%) (*p* = 0.02). Only 5 out of 36 symptomatic children also reported some other type of contact (3 at school, 1 elsewhere, 1 within the family) (Table 3).

**Table 3:** Reasons for performing a SARS-CoV-2 NS according to age-group

Reasons	Nursery school		Primary school		Secondary school		*P*	*P* for trend
	n	%	n	%	n	%		
N	46		62		54			
School contact (all)	11	23.9	32	51.6	27	50.0	0.008	0.011
Contact with a positive teacher	5	10.9	13	21.0	5	9.3	0.147	0.745
Contact with a positive child	3	6.5	14	22.6	16	29.6	0.014	0.005
Flu symptoms	13	28.3	13	21.0	10	18.5	0.483	0.251
Household contact with a positive case	17	37.0	12	19.4	8	14.8	0.022	0.010
Household contact with a contact of a positive case	6	13.0	6	9.7	2	3.7	0.237	0.095
Contact with a positive case in a location other than school or home	6	13.0	5	8.1	10	18.5	0.235	0.378

Nursery school: 0–5 years; Primary school: 6–10 years; Secondary school: 11–19 years

None of the controls reported more than one reason, while 15 (18.5%) cases reported more than one reason, with 14 reporting 2 reasons and 1 reporting 3 reasons (*p* < 0.001). A multivariable logistic regression with robust errors was performed to assess the probability of being Sars-CoV-2 positive in children reporting one reason (*n* = 147; 66 cases and 81 controls) (Fig. 1). No differences in the probability of being positive in children who were tested due to a household contact with a positive subject or a contact of a positive subject compared to those who were tested due to a contact with a positive subject in another location. The probability of being positive was significantly lower in children who had a school contact (OR = 0.19; 95% CI: 0.07–0.50) or who presented flu symptoms in absence of any known contact (OR = 0.17; 95% CI: 0.06–0.52). Similar results were obtained from both the logistic regressions adjusted for sex and age-class and the conditional logistic regression (Table 1 in Supplemental content).

**Fig. 2:**
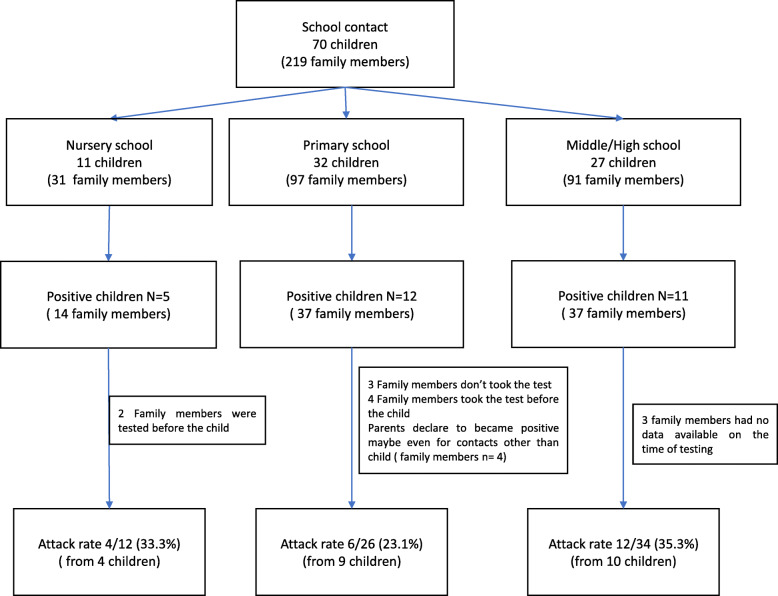
Transmission of SARS-CoV-2 infection from children (28 cases and 42 controls) who tested positive to a NS performed due to school contact to their family members

To assess the possible role of children in the spreading of the infection, we analyzed the number of family members of positive children that were infected at school, who subsequently resulted as positive for SARS-CoV-2. Twenty-eight out of 70 children resulted as SARS-CoV-2 positive following a school contact, thus leading to 88 at-risk family members. Of these, data from 16 family members were excluded as their NS was missing or was performed before the children’s NS, or because they declared being positive due to a different contact than their child. A total of 22 secondary cases were found among the 72 considered family members, with a secondary attack rate of 30.6% (95% CI: 20.2–42.5). The age-specific attack rates were similar for nursery, primary and middle/high school children (33.3% vs 23.1% vs 35.3%, respectively) (Fig. 2).

All symptomatic children within our study population presented a mild form of the disease, and none of them needed to be hospitalized. At the time of the SN, 15 (18.5%) positive cases and 21 (25.9%) controls presented at least one symptom (*p* = 0.25). The frequency of most of the symptoms, including fever and cough, did not differ between cases and controls, while gastrointestinal symptoms and rhinitis were significantly more frequent in controls than in positive cases. Within 14 days from the NS, however, almost half of the SARS-CoV-2 positive children (46/81; 56.8%) either developed or continued to show at least one symptom (fever, cough, rhinitis, headache, anosmia, gastrointestinal symptoms, asthenia) with a significantly higher frequency than controls (2/81; 2.5%, *p* < 0.001) (Table 3 in Supplemental content).

The 80% (4/5) of symptomatic children at the time of the NS spread the virus to their family members compared to 26.7% (18/67) of asymptomatic children (*P* = 0.028). The attack rate in symptomatic children was 36.6% (15/41), while the attack rate in asymptomatic children was 22.6% (7/31) (*P* = 0.201).

**Fig. 3:**
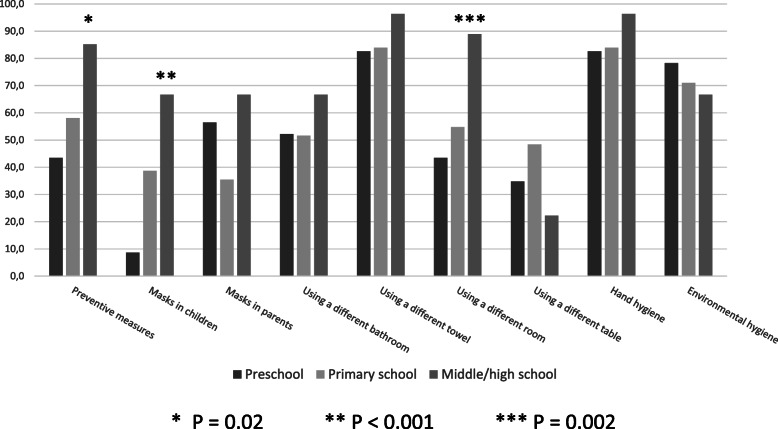
Percentage of children/family members applying recommended preventive measures according to type of measure and age group

The adherence to preventive measure against the spreading of SARS-CoV-2 was lower in the families of positive children. Only 63% of the family members implemented preventive measures, with parents of nursery school children being the ones who took significantly less precautions, in particular when considering the use of face masks both in children (8.7% vs 38.7 in primary and 66.7% in middle/high school) and in parents (56% vs 35% in primary and 66,7% in middle/high school) (Fig. 3).

## Discussion

According to the WHO [1], children who tested positive for SARS-CoV-2 acquired the infection more frequently at home, while only few outbreaks were reported in schools. However, these evidences derived for the most part from studies performed while school were closed and other lockdown measures were on [8]. Our study was carried out during the second wave of Covid-19 in Italy. Our results showed a lower probability of infection in children who had a school contact compared to children who had a household contact. The implemented procedures in classrooms, before the reopening of schools and the second wave in Italy may have contributed [21, 22], as suggested by an Italian study conducted in two school complexes of Rome in the period 21th september-4th December suggested [23]. This confirms that house-contacts are the main reason for SARS-CoV-2 infection, with 35.8% SARS-CoV-2 positive subjects reporting a contact with a positive case within the household and 14.8% reporting contact with a contact of positive within household, compared to 34.6% of SARS-CoV-2 positive cases reporting a school-contact.

The role of children in the transmission of SARS-CoV-2 is still unclear. According to the WHO, younger children, such as nursery school and primary school children seem to be less involved in the transmission of SARS-CoV-2 when compared to adolescents and adults [1]. A high heterogeneity across studies was reported in relation to the transmission of the infection from children to the other family members within the household. Several studies reported few cases of family clusters where children were the index cases [24–26]. Two more studies carried out in Greece [27] and South Korea [28] also reported that children were very rarely a cause of secondary transmission of the infection. However, another study carried out in Korea reported positive cases in 11.8% of household contacts [29], and a recent study performed in the US concluded that the transmission of the infection within the household is common, even by children. The study reported an overall secondary infection rate of 53% and a secondary attack rate of 53% (95% CI: 31–74%), with index cases being aged < 12 years [30]. Children who resulted as positive due a school contact in our population had high proportion of family members who subsequently resulted as positive, thus leading to a secondary attack rate of 30.6% (95% CI 20.2–42.5). Moreover, the infection rate, while being similar across the 3 age groups, resulted higher among symptomatic children, in particular within the first few days after the onset of symptoms. Even if this could be explained by a peak of respiratory viral load immediately after symptom onset, followed by a rapid decline in children [31] the observation may even derive from other factors (low number of children, different adherence to preventive measures, etc).

Three studies carried out in Germany [32], Australia [ 33] and France [34] report that the transmission of SARS-CoV-2 infection in the school setting or in the kindergarden [35] seems to be relatively uncommon, even though some studies did not consider asymptomatic cases [36]. Two Italian studies carried out at the begin of the second wave (September–October 2020) showed a low transmission of SARS-CoV-2 infection among students. In the first, the transmission of SARS-CoV-2 in 41 classes of 36 schools in northern Italy showed an overall secondary attack rate of 3.2%, that reached 6.6% in middle and high schools [37]. The second study showed a total of 1350 cases of SARS-CoV-2 infections in the Italian territory schools, involving 1050 students, for a total of 1212 out of 65,104 (1.8%) Italian schools involved [38]. A study carried out in the USA showed that reducing school attendance to only 2 days per week in combination with strict preventive measures allowed to significatively limit the secondary transmission of SARS-CoV-2 within the schools [39]. Our study was carried out in a more advanced period, during the peak of the second wave (October–December 2020) and, even though school contact was the most frequent reason to do a NW, the probability of being positive resulted almost 5 times lower in children who reported a school contact compared to children who reported a household contact (OR 0.19; 95% CI: 0.07–0.50). (Fig. 1).

A recent retrospective database cohort study conducted in a large Health Maintenance Organization in Israel showed that 602 (58.3%) out of 1032 children were infected by a parent and 122 (11.8%) acquired the infection at school [ 40] and only 6 (5%) of the latter resulted in secondary cases in household. The Authors conclude that children are less likely to be the vector for the infection within the household. However, this study include only a relatively short time since schools were re-opened during a period of low infection rate. Finally, a multicenter prospective study in Spain performed during the summer and school periods (1 July-31 October, 2020), during the second wave, showed that 7.7% (80/1040) of pediatric cases were index cases, with a secondary attack rates of 59%. Only a slight unsignificant difference was showed between the pediatric index cases between summer (7.1%) and school period (8.3%) was showed [41].

In our study, conducted during the second wave of the SARS-CoV-2 infection, 28 children who acquired the infection at school resulted in 22 secondary cases, with a secondary attack rate of 30.6%. Differences in the implementation of the preventive measures against the spreading of the infection at home or a diverse variant of SARS-CoV-2, could explain the different results.

The WHO recommended a set of measures to limit the spreading of the infection, with a particular focus on individual measures, including frequent hand hygiene, social distancing, respiratory etiquette, use of face masks and PPEs if symptomatic or when caring for symptomatic cases [42]. Results from different publications showed that the percentage of adherence to these preventive measures outside the households varied across studies, ranging from 67 to 72% [ 43] to 86–90% [44]. The variability might be due to several factors, including differences in the economic and educational level [45]. Few studies investigated the adherence to preventive measures at home in case of symptomatic children. When considering the ten recommended quarantine measures, Lou showed that the proportion of families who followed those measures and kept a 1.5 m distance, practiced proper hand hygiene, wore face masks at home, and applied a proper cough etiquette was very low (< 30% for each measure) [46]. A study by Yun et al. showed that the risk of infection of the caregivers in presence of symptomatic children was lower when the caregiver reported using a face mask and practicing hand hygiene, with no positive case among 15 caregivers of children with mild Covid-19 symptoms [47]. Our study showed that, despite the 1-year length of the Covid-19 pandemic, and the subsequent continuous dissemination of information on effective preventive measures through all media, the adherence to many of such measures at home is still low, in particular in case of symptomatic children of preschool age.

The present study has some limitations, mainly due to its retrospective design. In particular, a degree of recollection bias can be assumed on information relating to symptoms and other data. However, the time between the swab and the interview was usually very short, and the interviewed parents were very motivated to answer, as they felt that the reason for administering the questionnaire was very relevant to them.

After the first wave, a debate over school reopening has been developed. Due to report about children harms such as learning delay, increased mental health concerns, increased child abuse, etc., as well as of their negative economic impact, the balance between benefit and harms seems shift against school closure [48–50]. The WHO stated that children and schools are unlikely to be the main drivers of SARS-CoV-2 transmission, when community transmission is low and when appropriate mitigation measures are applied [1]. In a recent publication, Munro requested the schools to be reopened, reporting that children are not super spreaders [51]. However, the probability of infection seem to significantly increase when the community transmission rate is higher. According to Klimek-Tulwin et al. and to Wang et al., during the first wave of the pandemic, a delay in closing the schools was significantly associated to an increase in the incidence rate of infection during the following days [52, 53].

Furthermore, during the second wave, Ferretti et al. reported that the incidence rate among secondary school students almost doubled when compared to the general population of the same region [54]. However, the topic remains debated given that other studies have shown opposite results, as reported in a recent review on this topic [55]. A Spanish multicenter study, conducted during the second wave, led the authors to conclude that children do not contribute much to family clusters even when school are opened [41]. Similar conclusions have been showed in a recent Italian study, performed during the second wave, that did not show any relationship between the opening of the schools and an increase in the transmission rate (RT) index in the general population [56].

In conclusion, our study showed that during the second wave of the pandemic school contact was the most frequent reason to do a NW. However, the risk of being positive to SARS-CoV-2 was lower after a school contact when compared to a household contact thus both situations contribute in a similar way (35,8 vs 34,6%) to the spreading of SARS-CoV-2 infection in children.

Children of any age group can spread the infection to their family members at home and in nursery school children this may be due to a lower adherence to preventive measures at home. Additional initiatives aimed to increase awareness of the significant contagiousness of children at home and therefore of the need of joining preventive measures at home are needed, to motivate parents and family members to consistently implement these measures, in particular when is know that the child had been in contact with a positive case.

## Supplementary Information

**Additional file 1.** .

## Data Availability

The datasets used and7or analysed during the current study are available from the corresponding author on reasonable request.

## Abbreviations

SARS-CoV-2: Severe Acute Respiratory Syndrome Coronavirus 2
COVID-19: Coronavirus Disease 2019
SCPWC: S. Camillo-Forlanini Pediatric Walk-in Center
NS: Nasopharyngeal swabs
Ag RDT: Antigen rapid detection test
COI: Cut off index
NAAT: Nucleic acid amplification test
OR: Odds Ratio
CI: Confidence Interval
WHO: World Health Organization

## References

[1] WHO What we know about COVID-19 transmission in schools Coronavirus Update 39, 21 October 2020. http://www.who.int/emergencies/diseases/novel-coronavirus-19/question-and-answer-hub/q-a-detail/coronavirus-disease-covid-19-schools.

[2] Katsuta T, Shimizu N, Okada K, Tanaka-Taya K, Nakano T, Kamiya H, et al. The clinical characteristics of pediatric coronavirus disease 2019 in 2020 in Japan. Pediatr Int. 2021. 10.1111/ped.14912 Epub ahead of print. PMID: 34233075. 10.1111/ped.14912 PMC8446955 34233075

[3] European Centre for Disease Prevention and Control. COVID-19 in children and the role of school settings in transmission - first update. Stockholm; 2020. European Center for Disease Prevention and Control. Technical Report.

[4] Goldstein E, Lipsitch M (2020). On the effect of age on the transmission of SARS-CoV-2 in households, schools and the community. medRxiv 2020.07.19.20157362.

[5] Viner RM, Mytton OT, Bonell C, Melendez-Torres GJ, Ward J, Hudson L, Waddington C, Thomas J, Russell S, van der Klis F (2021). Susceptibility to SARS-CoV-2 Infection Among Children and Adolescents Compared With Adults A Systematic Review and Meta-analysis. JAMA Pediatr.

[6] Rota MC, Bellino S, Vescio MF, et al. Opening of schools and progress confirmed cases of SARS-CoV-2: the situation in Italy. ISS Covid 19 report. No 63/2020. https://www.iss.it/rapporti-covid-19.

[7] Park YJ, Choe YJ, Park O, Park SY, Kim YM, Kim J, Kweon S, Woo Y, Gwack J, Kim SS, Lee J, Hyun J, Ryu B, Jang YS, Kim H, Shin SH, Yi S, Lee S, Kim HK, Lee H, Jin Y, Park E, Choi SW, Kim M, Song J, Choi SW, Kim D, Jeon BH, Yoo H, Jeong EK, on behalf of the COVID-19 National Emergency Response Center, Epidemiology and Case Management Team (2020). Contact tracing during coronavirus disease outbreak, South Korea, 2020. Emerg Infect Dis.

[8] COVID-19 in children and the role of school settings in COVID-19 transmission, 6 August 2020. Stockholm: ECDC; 2020. European Center For Disease Prevention and Control.

[9] Li X, Xu W, Dozier M, He Y, Kirolos A, Theodoratou E, on behalf of UNCOVER (2020). The role of children in transmission of SARS-CoV-2: A rapid review. J Global Health.

[10] World Health Organization. WHO Director—General’s Openings Remarks at the Media Briefing on COVID-19. 11 March 2020. Available online: https://www.who.int/director-general/speeches/detail/whodirector-general-s-opening-remarks-at-the-media-briefing-on-covid-19%2D%2D-11-march-2020

[11] Decreto del Presidente del Consiglio dei Ministri del 11 marzo 2020. GU Serie Generale n.64 del 11-3-2020 https://www.gazzettaufficiale.it/eli/id/2020/03/11/20A01605/sg

[12] Decreto del Presidente del Consiglio dei Ministri del 7 settembre 2020. GU Serie Generale n.222 del 07-09-2020. https://www.gazzettaufficiale.it/eli/id/2020/09/07/20A04814/sg

[13] ISSN Epicentro. https://www.epicentro.iss.it/coronavirus/. Accessed 6 Aug 2021.

[14] Johns Hopkins University &Medicine. Coronavirus Resource Center. https://coronavirus.jhu.edu/map.html. Accessed 6 Aug 2021.

[15] Wallace M, Woodworth KR, Gargano JW, Scobie HM, Blain AE, Moulia D, Chamberland M, Reisman N, Hadler SC, MacNeil JR, Campos-Outcalt D, Morgan RL, Daley MF, Romero JR, Talbot HK, Lee GM, Bell BP, Oliver SE (2021). The advisory committee on immunization practices’ interim recommendation for use of Pfizer-BioNTech COVID-19 vaccine in adolescents aged 12–15 years—United States, may 2021. MMWR Morb Mortal Wkly Rep.

[16] Scherer AM, Gedlinske AM, Parker AM, Gidengil CA, Askelson NM, Petersen CA, Woodworth KR, Lindley MC (2021). Acceptability of Adolescent COVID-19 Vaccination Among Adolescents and Parents of Adolescents - United States, April 15-23. MMWR Morb Mortal Wkly Rep.

[17] Saxena S, Skirrow H, Wighton K (2021). Should the UK vaccinate children and adolescents against covid-19? The UK is an outlier in holding off vaccinating healthy 12-17 year olds. BMJ.

[18] Hughes B, Miller-Idriss C, Piltch-Loeb R, Goldberg B, White K, Criezis M, Savoia E (2021). Development of a Codebook of Online Anti-Vaccination Rhetoric to Manage COVID-19 Vaccine Misinformation. Int J Environ Res Public Health.

[19] Ministero della Salute, OGGETTO: Aggiornamento della definizione di caso COVID-19 e strategie di testing. 0000705–08/01/2021-DGPRE-DGPRE-P. http://www.salute.gov/portale/nuovocoronavirus/.

[20] European Centre for Disease Prevention and Control (2020). Options for the use of rapid antigen tests for COVID-19 in the EU/EEA and the UK.

[21] Cento V, Alteri C, Merli M, Di Ruscio F, Tartaglione L, Rossotti R, et al. Effectiveness of infection-containment measures on SARS-CoV-2 seroprevalence and circulation from May to July 2020, in Milan, Italy. PLoS One. 2020;15(11):1-12. 10.1371/journal.pone.0242765 PMC7679019 33216817

[22] Villani A, Bozzola E, Siani P, Corsello G (2020). The Italian pediatric society recommendations on children and adolescents extra-domestic activities during the SARS-CoV 2 emergency phase 2. Ital J Pediatr.

[23] Villani A, Coltella L, Ranno S (2021). Bianchi di Castelbianco F, Murru PM, Rossella Sonnino6, Teresa Mazzone2,7, Livia Piccioni3, Giulia Linardos3, Stefano Chiavelli3, Fabrizio Pontarelli1, Giovanni Corsello2,8, Massimiliano Raponi9, Carlo Federico Perno3* and Carlo Concato3: School in Italy: a safe place for children and adolescents. Ital J Pediatr.

[24] Teherani MF, Kao CM, Camacho Gonzalez A (2020). Burden of Illness in Households With Severe Acute Respiratory Syndrome Cor onavirus 2 Infected Children. J Pediatric Infect Dis Soc.

[25] Somekh E, Gleyzer A, Heller E (2020). The Role of Children in the Dynamics of Intra Family Coronavirus 2019 Spread in Densely Populated Area. Pediatr Infect Dis J.

[26] Lee EJ, Kim DH, Chang SH, Suh SB, Lee J, Lee H, Han MS (2021). Absence of SARS-CoV-2 transmission from children in isolation to guardians, South Korea. Emerg Infect Dis.

[27] Maltezou HC, Vorou R, Papadima K, Kossyvakis A, Spanakis N, Gioula G, Exindari M, Metallidis S, Lourida AN, Raftopoulos V, Froukala E, Martinez-Gonzalez B, Mitsianis A, Roilides E, Mentis A, Tsakris A, Papa A (2021). Transmission dynamics of SARS CoV 2 within families with children in Greece: a study of 23 clusters. J Med Virol.

[28] Kim J, Choe YJ, Lee J, et al. Role of children in household transmission of COVID 19. Arch Dis Child. 10.1136/archdischild-2020-319910 Online ahead of print. 10.1136/archdischild-2020-319910 32769089

[29] Park YJ, Choe YJ, Par k O (2020). Contact Tracing during Coronavirus Disease Outbreak, South Korea, 2020. Emerg Infect Dis.

[30] Grijalva CG, Rolfes MA, Zhu Y, McLean H, Hanson KE, Belongia EA, Halasa NB, Kim A, Reed C, Fry AM, Talbot HK (2020). Transmission of SARS COV 2 infections in households Tennessee and Wisconsin, April September 2020. MMWR Morb Mortal Wkly Rep.

[31] Jones TC, Muhlemann B, Veith T, et al. An analysis of SARS-CoV-2 viral load by patient age. MedRxiv preprint. 10.1101/2020.06.08.20125484.

[32] Otte Im Kampe E, Lehfeld AS, Buda S, Buchholz U, Haas W (2020). Surveillance of COVID 19 school outbreaks, Germany, March to August 2020. Euro Surveill.

[33] Macartney K, Quinn HE, Pillsbury AJ (2020). Transmission of SARS CoV 2 in Australian educational settings: a prospective cohort study. Lancet Child Adolesc Health.

[34] Danis K, Epaulard O, Bénet T (2020). Cluster of coronavirus disease 201 9 (Covid 19) in the French Alps, 2020. Clin Infect Dis.

[35] Thielecke M, Theuring S, van Loon W, et al. SARS-CoV-2 infections in kindergartens and associated households at the start of the second wave in Berlin, Germany – a cross sectional study. Eur J Public Health. 2021:ckab079. 10.1093/eurpub/ckab079 Online ahead of print. 10.1093/eurpub/ckab079 PMC8135989 33956945

[36] Heavey L, Casey G, Kelly C, Kelly D, McDarby G (2020). No evidence of secondary transmission of COVID 19 from children attending school in Ireland, 2020. Euro Surveill.

[37] Larosa E, Djuric O, Cassinadri M, Cilloni S, Bisaccia E, Vicentini M, Venturelli F, Giorgi Rossi P, Pezzotti P, Bedeschi E, the Reggio Emilia Covid-19 Working Group (2020). Secondary transmission of COVID 19 in preschool and school settings in northern Italy after their reopening in September 2020: a population based study. Euro Surveill.

[38] Buonsenso D, De Rose C, Moroni R, Valentini P. SARS-CoV-2 Infections in Italian Schools: Preliminary Findings After 1 Month of School Opening During the Second Wave of the Pandemic. Front Pediatr. 8:615894. 10.3389/fped.2020.615894. 10.3389/fped.2020.615894 PMC7841339 33520898

[39] Zimmerman KO, Akimboyo IC, Brookhart MA (2021). Incidence and secondary transmission of SARS-CoV-2 infections in schools. Pediatrics.

[40] Shapiro Ben David S, Cohen D, Tasher D, Geva A, Azuri J, Ash N. COVID-19 in children and the effect of schools reopening on potential transmission to household members. Acta Paediatr. 2021. 10.1111/apa.15962 Online ahead of print. PMID: 34053108. 10.1111/apa.15962 PMC8222890 34053108

[41] Soriano-Arandes A, Gatell A, Serrano P, Biosca M, Campillo F, Capdevilla R, et al. Household SARS-CoV-2 transmission and children: a network prospective study. Clin Infect Dis. 2021:ciab228. 10.1093/cid/ciab228 PMCID: PMC79895. 10.1093/cid/ciab228 PMC7989526 33709135

[42] World Health Organisation. Overview of public health and social measures in the context of COVID-19: interim guidance, 2020: World Health Organization; 2020. Available from: https://www.who.int/publications/i/item/overview-of-public-health-and-social-measures-in-the-context-of-covid-19.13.

[43] Block R, Berg A, Lennon RP, Miller EL, Nunez-smith M (2020). African American adherence to COVID-19 public health recommendations. Health Lit Res Pract.

[44] Lennon RP, Sakya SM, Miller EL, Snyder B, Yaman T, Zgierska AE, Ruffin MT, Van Scoy LJ (2020). Public intent to comply with COVID-19 public health recommendations. Health Lit Res Pract.

[45] Weiss BD, Paasche Orlow MK. Disparities in Adherence to COVID-19 Public Health Recommendations. Health Lit Res Pract. 2020;4(3). 10.3928/24748307-20200723-01 PMC7410493 32926173

[46] Lou Q, Su DQ, Wang SQ, Gao E, Li LQ, Zhuo ZQ (2020). Home quarantine compliance is low in children with fever during COVID-19 epidemic. World J Clin Cases.

[47] Yun KW, Kim KM, Kim YK, Kim MS, Kwon H, Han MS, Lee H, Choi EH (2021). Limited benefit of facility isolation and the rationale for home Care in Children with mild COVID-19. J Korean Med Sci.

[48] Dooley DG, Christakis D (2021). It is time to end the debate over School reopening. JAMA Netw Open.

[49] Bekkering G, Delvaux N, Vankrunkelsven P, Toelen J, Aertgeerts S, Crommen S, De Bruyckere P, Devisch I, Lernout T, Masschalck K (2021). Closing schools for SARS-CoV- 2: a pragmatic rapid recommendation. BMJ Paediatr Open.

[50] Buonsenso D, Roland D, De Rose C, Vasquez-Hoyos P, Ramly B, Chakakala-Chaziya JN, Munro A, Gonzalez-Dambrauskas S (2021). Schools closures during the COVID-19 pandemic. A catastrophic global situation. Ped Infect Dis J.

[51] Munro APS, Faust SN (2020). Children are not COVID-19 super spreaders: time to go back to school. Arch Dis Child.

[52] Klimek-Tulwin M, Tulmin T (2020). Early school closures can reduce the first-wave of the COVID-19 pandemic development. Z Gesundh Wiss.

[53] Wang X, Pasco RF, Du Z, Petty M, Fox SJ, Galvani AP, et al. Impact of Social Distancing Measures on Coronavirus Disease Healthcare Demand, Central Texas, USA. Emerg Infect Dis. 2020;26(10) *www.CDC.Gov/eid*. 10.3201/eid2510.201702. 10.3201/eid2610.201702 PMC7510701 32692648

[54] Ferretti, A. Cade il Velo sui Contagi nelle Scuole Piemontesi: Il Personale da due a Quattro Volte Più Esposto della Media, la Situazione Nella Scuola Dell’infanzia è Drammatica. Solo le Superiori si Salvano Grazie alla DAD, 7 December 2020. Available online: https://alessandroferrettiblog.wordpress.com/2020/12/07/cade-il-velo-sui-contagi-nelle-scuole-piemontesiil- personale-da-due-a-quattro-volte-piu-esposto-della-media-la-situazione-nelle-materne-e-drammatica-solo-le-superiori-sisalvano- grazie-alla-dad/ (accessed on 29 March 2021).

[55] Busa F, Barzanellu F, Pintus MC, Fanos V, Marcialis MA. COVID-19 and school: to open or not to open, That Is the Question. The First Review on Current Knowledge. Pediatr Rep. 2021;13(2):257-278. 10.3390/pediatric13020035 PMC8293384 34205837

[56] Gandini S, Rainisio M, Iannuzzo ML, Bellerba F, Cecconi F, Scorrano L (2021). A cross-sectional and prospective cohort study of the role of schools in the SARS-CoV-2 second wave in Italy. Lancet Reg Health Eur.

