# Supplementary material for: Reasons for SARS-CoV-2 infection in children and their role in the transmission of infection according to age: a case-control study
Source: Ital J Pediatr. 2021 Sep 27;47:193. doi: 10.1186/s13052-021-01141-1 (PMC8474731; doi:10.1186/s13052-021-01141-1)
Supplement: Supplementary file 1 — Additional file 1. . [file 13052_2021_1141_MOESM1_ESM.docx]

**Questionnaire**

**General questions**

1. Why did you test the child?
2. Were any of your household positive for SARS-CoV-*2* or affected from COVID-19?
3. Have you been in contact with someone positive for SARS-CoV-2 or affected from COVID-19, hence you test the child?
4. Has the child been in contact with a positive case at school?
5. Was this positive contact a child?
6. Was this positive contact a teacher?
7. Other reasons?
8. Did you take the test too?
9. Have any of your households tested positive?
10. If yes, before or after the child?
11. If later, how many days after?
12. Do you think that your son infected you or you both have been in contact with a positive case?
13. Does the child go to school?
14. If yes, which one ?
15. How many people do you live in the house with?
16. Who is the child's family pediatrician?

**Questions about how the child was in the days following the test**

1. In the days following taking the test, how was the child?
2. Well? Sick? What symptoms did he have? Cold, cough, phlegm, anosmia, diarrhea, etc.
3. Was he hospitalized?
4. Did you then repeat SARS-CoV-2 test after the first test again?
5. If yes, after how many days and what was the result?

**Questions about the preventive measures put in place as soon as they knew that the child has been in contact with a positive**

1. What preventive measures did you use as soon as you knew that the child could have been in contact with a positive?
2. Did the child wear a surgical mask at home?
3. Did family members wear a surgical mask at home?
4. Did the child use a separate bathroom?
5. Did the child use a disposable towel or a personal towel?
6. Did the child sleep in a separate room?
7. Did the child eat at the table with you?
8. Did you wash your hands after a contact with the child?
9. Did you disinfected the surfaces in your home every day?

Table 1 in Supplemental content: Multivariable logistic regression to evaluate the influence of reason for running SARS-CoV-2 NS on positive results.

|  | model 1 | | | model 2 | | | model 3 | | | model 4 | | |
| --- | --- | --- | --- | --- | --- | --- | --- | --- | --- | --- | --- | --- |
| N | aOR | 95% CI | p | aOR | 95% CI | p | aOR | 95% CI | p | OR | CI95% | p |
|  |  |  |  |  |  |  |  |  |  |  |  |  |
| Household contact with a positive case | 1 |  |  | 1 |  |  | 1 |  |  | 1 |  |  |
| Household contact with a contact of a positive case | 0.73 | 0.11-4.80 | 0.741 | 0.76 | 0.12-4.86 | 0.768 | 0.74 | 0.06-9.00 | 0.810 | 0.73 | 0.11-4.77 | 0.74 |
| School contact | 0.19 | 0.07-0.50 | 0.001 | 0.16 | 0.06-0.45 | 0.001 | 0.14 | 0.04-0.48 | 0.002 | 0.19 | 0.07-0.51 | 0.001 |
| Flu symptoms | 0.17 | 0.06-0.52 | 0.002 | 0.16 | 0.05-0.49 | 0.001 | 0.15 | 0.04-0.55 | 0.004 | 0.17 | 0.06-0.54 | 0.003 |
| Contact with a positive case in a location other than school or home | 0.36 | 0.10-1.30 | 0.120 | 0.35 | 0.09-1.36 | 0.129 | 0.41 | 0.09-1.69 | 0.218 | 0.36 | 0.10-1.37 | 0.136 |
| ^Multivariable logistic regression | |  |  |  |  |  |  |  |  |  |  |  |
| model 1=unconditional logistic regression robust errors (all variables in the table n=147) | | | | | | |  |  |  |  |  |  |
| model 2= unconditional logistic regression robust errors (all variables adjusted for sex and class of age n=147) | | | | | | | | | |  |  |  |
| model 3= conditional logistic regression (n=132) | | |  |  |  |  |  |  |  |  |  |  |
| model 4 = unconditional logistic regression clustered errors (pair) (all variable, n=147) | | | | | | | |  |  |  |  |  |

Table 2 Supplemental content: Demographic data of the study population

|  | COVID-19 positive |  | Controls |  |
| --- | --- | --- | --- | --- |
|  | n | % | n | % |
| All | 81 |  | 81 |  |
| **Sex** |  |  |  |  |
| Male | 44 | 54.3 | 44 | 54.3 |
| Female | 37 | 45.7 | 37 | 45.7 |
| **Age** |  |  |  |  |
| Media (sd) | 8.6 | 4.5 | 8.7 | 4.6 |
| Nursery school  0-5 anni | 23 | 28.4 | 23 | 28.4 |
| Primary school  6-10 | 31 | 38.3 | 31 | 38.3 |
| Middle/high school  11-19 | 27 | 33.3 | 27 | 33.3 |

Nursery school:0-5 years; Primary school: 6-10 years; Middle/high school: 11-19 years

Age- and sex-matched consecutive children with a SARS-CoV-2 NS negative result were selected as controls

Table 3 Supplementary material: Symptoms at the time of performing the SARS-CoV-2 NS and throughout the following 2 weeks

|  | Pre (When the NS was done) | | | | | Post (in the 2 weeks after the NS) | | | | |
| --- | --- | --- | --- | --- | --- | --- | --- | --- | --- | --- |
|  | Covid-19 - | | Covid-19 + | | p | Covid-19  - | | Covid-19 + | | p |
|  | n | % | n | % |  | n | % | n | % |  |
| N | 21 |  | 15 |  |  | 2 |  | 46 |  |  |
| Fever | 15 | 71.4 | 14 | 93.3 | 0.102 | 0 | 0.0 | 27 | 58.7 | 0.186 |
| Cough | 1 | 4.8 | 0 | 0.0 | 0.391 | 0 | 0.0 | 5 | 10.9 | 0.999 |
| Rhinitis | 7 | 33.3 | 1 | 6.7 | 0.058 | 2 | 100.0 | 9 | 19.6 | 0.049 |
| Anosmia | 0 | 0.0 | 1 | 6.7 | 0.230 | 0 | 0.0 | 10 | 21.7 | 0.999 |
| Ageusia | 0 | 0.0 | 0 | 0.0 |  | 0 | 0.0 | 2 | 4.3 | 0.999 |
| Gastrointestinal | 7 | 33.3 | 0 | 0 | 0.013 | 0 | 0.0 | 6 | 13.0 | 0.999 |
| Musculoskeletal | 0 | 0.0 | 0 | 0.0 |  | 0 | 0.0 | 0 | 3.0 | 0.999 |
| Headache | 0 | 0.0 | 1 | 6.7 | 0.230 | 0 | 0.0 | 8 | 17.4 | 0.999 |
| Asthenia | 0 | 0.0 | 0 | 0.0 |  | 0 | 0.0 | 7 | 15.2 | 0.999 |

Post: percentage was calculated on patients with symptoms
